# Loss and recovery after concussion: Adolescent patients give voice to their concussion experience

**DOI:** 10.1111/hex.13138

**Published:** 2020-10-07

**Authors:** Romita Choudhury, Ash Kolstad, Vishvesh Prajapati, Gina Samuel, Keith Owen Yeates

**Affiliations:** ^1^ Patient and Community Engagement Research Program O'Brien Institute of Public Health Cumming School of Medicine University of Calgary Calgary AB Canada; ^2^ Sport Injury Prevention Research Centre Faculty of Kinesiology University of Calgary Calgary AB Canada; ^3^ Ronald and Irene Ward Chair in Pediatric Brain Injury Department of Psychology University of Calgary Calgary AB Canada

**Keywords:** adolescent health, concussion, coping, mental health, patient education, patient engagement, patient experience, recovery, resilience

## Abstract

**Background:**

Most concussion studies have focused on the perspectives and expertise of health‐care providers and caregivers. Very little qualitative research has been done, engaging the adolescents who have suffered concussion and continue to experience the consequences in their everyday life.

**Objective:**

To understand the experiences of recovery from the perspective of adolescent patients of concussion and to present the findings through their voices.

**Methods:**

Two semi‐structured focus groups and two narrative interviews were conducted with a small group of 7 adolescents. Grounded theory was used to analyse the data.

**Results:**

Participants experience continuing difficulty 1‐5 years after treatment with cognitive, emotional, social and mental well‐being. The overriding experience among older adolescents (17‐20) is a sense of irreversibility of the impact of concussion in all these areas.

**Conclusion:**

There is a significant gap between the medical determination of recovery and what patients understand as recovery. Adolescents do not feel ‘recovered’ more than a year after they are clinically assessed as ‘good to go’. Systematic follow‐up and support from a multi‐disciplinary health‐care team would strengthen youths' coping and resilience.

## 1 INTRODUCTION

Although concussions are common among adolescents aged 13‐19 years and the highest rate of injuries are reported in the under‐19 age group,^1^, ^2^ very little qualitative research exists on the experience of adolescents and their means of coping with what might be a life‐altering injury.^3^, ^4^ In Canada, 46 000 concussions in the age group of 5‐19 years presented at hospital emergency departments in years 2016‐17.^5^ According to the Canadian Hospitals Injury Reporting and Prevention Program (CHIRPP) report,^6^ the highest proportion of brain injuries among children and youth 5‐19 years of age occurred in ice hockey, rugby and ringette, constituting 27%‐44% of all injuries that happened while playing these sports. From a clinical perspective, most adolescents would be considered recovered within 1‐4 weeks after injury.^7^ Yet, many continue to experience the impact of concussion for months, even years, after they are deemed fit to return to school and play, especially after multiple injuries.^8^ Evidence suggests that high school students take longer to recover from concussions than younger children and adults.^9^, ^10^ Both physiological and psychological factors have been recognized as significant in the actual recovery time of youth, high school and collegiate athletes.^11^ Reportedly, ‘the latest Concussion in Sports Group (CISG) guidelines state that athletes should return to a baseline level of symptoms but do not provide definitions to establish when an athlete is fully recovered physiologically and ready to return to sport’ (p.2).^12^ Emerging and more comprehensive measures of sports‐related concussion impact show that children are likely to experience a gamut of long‐term neurophysiological outcomes from cognitive and mental health problems to loss in vision, hearing, motor and sensory abilities.^13^

Studies of quality of life in adolescents with concussion show that the cognitive, emotional and social life of these youth can be adversely affected even 5 years after the injury, not only in severe, but also after mild or moderate TBI.^14^, ^15^ Prolonged and persistent post‐concussion symptoms may ‘actually be caused or maintained by factors beyond the neurobiology of the concussion’.^3^ Furthermore, repeated concussions from sport often resulting in increased susceptibility to injury, even after the athlete has withdrawn from sports, have severe effects on the overall health of the individual.

## 2 SCOPE AND METHOD

### 2.1 Objective

Our qualitative research was part of an internship programme run by the Patient and Community Engagement Research (PaCER) Program. In this programme, interns are trained in specific qualitative research methods that have been adapted to maximize engagement of patients and can lead to high impact research. Two of our co‐authors are graduates of the programme and have first‐hand experience of sports‐related concussion. Using their personal experiences, combined with the co‐design and research skills learned through PaCER, they were able to elicit valuable information from other patients through focus groups and interviews in a safe, peer and patient‐focused setting. The purpose of the study was to uncover the experiences of adolescents recovering from concussion with a view to understanding what is most important to the patients themselves in the path to recovery and to represent their reality in their own words as much as possible.

### 2.2 Recruitment

Recruitment strategies included posters in hockey arenas, soccer clubs and martial arts training facilities, Brain Injury Rehabilitation Clinic, Alberta Children's Hospital, and social media postings (Twitter). A total of 7 participants in the age group of 13‐19 were recruited. All were athletes. The age range of 13‐19 was determined on the basis of higher risk of concussion and the ability to give informed consent (see Table 1).

**TABLE 1:** Demographic information

Pseudonym	Sex	Age Y	Sources of injury	Number of Concussions	Time since treatment Y
Alice	Female	16	Soccer	2	1
Ron	Male	13	Hockey	2	1
Devon	Male	13	Hockey	4	2
Hank	Male	19	Hockey	9	2
Ray	Male	19	Hockey	6	2
Marge	Female	17	Ringette	7	1
Sarah	Female	18	Basketball	2	5

For minors (aged 13‐17 years), procedures recommended by the Tri‐Council Policy Statement^16^ and the University of Rochester's Guideline for Assessing Capacity to Consent in Children^17^ were followed. Thus, only those minors were included whose decision‐making capacity was sufficient. A regular consent procedure was used for participants who had reached the age of 18 years. Real names of participants were replaced with pseudonyms to uphold the study's confidentiality agreement.

The recruitment size was smaller than expected due to several issues. Teenagers often do not stop to read posters or announcements for a research study. In addition, it was difficult to approach them directly, having to rely on sports authorities, clinic leaders and parents to convey the purpose of our study. A few parents felt that having their child revisit their concussion‐related experiences might be detrimental to their health.

### 2.3 Data collection and analysis

**FIGURE 1:**
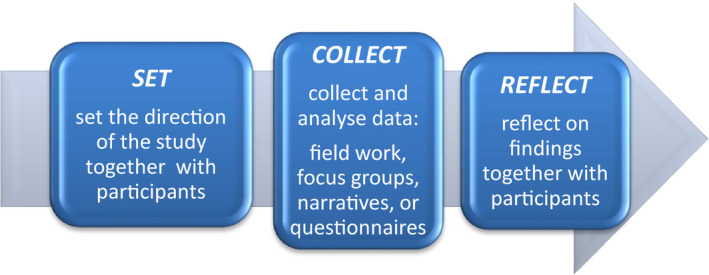
PaCER method

This qualitative research study combined a grounded theory thematic analysis framework^18^, ^19^ and constructivist narrative analysis technique.^20^ Using a distinct patient engagement structure defined as ***set, collect, reflect***, researchers ensured that patients were meaningfully engaged at every stage of the study^21^ (Figure 1). This is an iterative approach, driven by patients working with patients. It results in a shared collective understanding of the issue, one that is solidly grounded in patient experience. In this study, the *set* focus group clarified the scope and direction of the study, by identifying the issues that were of the highest importance to the patient participants and directed the guiding questions for the next phase. Data were then gathered, in what is called the *collect* phase, on those areas of concern identified by patients in the first phase, using focus groups and interviews. In the *reflect* phase, patients from the previous phases participated in a final focus group where they brought a common understanding to the *collect* findings and highlighted certain areas of their experience, such as mental health, better education for all involved, follow‐up and a more coordinated approach to treatment and recovery. All data were recorded and transcribed; codes were applied to segments of texts that corresponded to the themes in the focus groups and interviews. The codes were grouped into higher order categories and compared within the same coding category for similarities and differences.

The data that support the findings of this study are available from the corresponding author upon reasonable request.

## 3 RESULTS

### 3.1 Isolation after treatment

All patients underwent isolation and rest for varying amounts of time as part of their treatment. This was a difficult period and was described in metaphors of entrapment and emptiness. Far more difficult was the isolation after treatment which appeared to be an insurmountable part of life after concussion. All seven participants struggled in school, not just with catching up but with returning to pre‐concussion levels of aptitude, confidence, and sense of social ease and well‐being. Three areas that emerged as major contributors to their isolation were lower levels of comprehension, memory and concentration, all of which had a profound impact on their academic and social life.

#### 3.1.1 Comprehension

One recently graduated 18‐year‐old girl described her anguish at not being able to follow spoken and written words:

It took me very long to understand what people were saying. It took a long time to process things in my head and I had to think really really really hard. I felt I was brain damaged. That is kind of how I felt. (Sarah)

This perception of damage was echoed by another participant who felt so apprehensive about his ability to follow through with a conversation in a group environment that he learned to shun company altogether. The constant anxiety about comprehension created a sense ofinadequacy, compelling the adolescents to distance themselves from the groups to which they had belonged.

It is very rare that I have a conversation that I haven't already had multiple times in my head because there is such social anxiety about talking to people. (Hank)

I stress about what people are thinking… like are they judging me. I just don't feel as smart. I kind of lose my words. It is really bizarre. I never felt that before. (Sarah)

Marge explains her ‘personality changes’ by referring to the loss in vigour and enthusiasm.

I would always argue a point till the bitter end. I think I have become lazy.

This change presented itself as an abrupt phenomenon. It is as if everything familiar had suddenly been taken away in one fell swoop. None seemed prepared for this loss of certainty about things that they took for granted.

#### 3.1.2 Memory

Added to reduced comprehension is the challenge with remembering. Like comprehension, memory issues impacted both academic and social life. The continued struggle with memory well **after treatment** caused frustration and anxiety.

Group work is not bad cause it usually involves ideas coming from everyone. It is when I am trying to remember what I studied and that dialogue that is such a struggle. (Hank)

As I'm working, I am not really realizing what I'm doing right then but after a few minutes I would realize that when I built this file, I didn't put something in it. So then I'll write it in and then I'll completely forget again. (Sarah)

To the participants, the memory loss seemed permanent. The reassurances given by physicians that ‘it was probably going to go away with time’ seemed less and less realistic to them as they continued to struggle with what they described as one of the most difficult issues.

#### 3.1.3 Concentration

Combined with memory loss and slower comprehension, the struggle to concentrate imposed huge pressures both at school and play. Each one appeared to experience a specific type of challenge.

I can't concentrate when I'm alone. People talking can be helpful in the background but obviously not to me. I like to study back in the pool in the university because there's the sound of some people talking… that kinda overrides the headaches and forces me to focus on my school‐work more attentively instead of letting my mind wander. (Ray)

It is hard to concentrate now, with school. I have had MRI's done and I do have portions of my brain they have pointed out not working or they say dead cells or sections specifically to do with short term memory… for school work I have to repeat it so many times to engrave it and yeah I have to study for long periods of time, things like that. (Hank)

Although the adolescents in our study devised various ways of coping with their condition by working in isolation, taking copious notes or finding conducive settings, one could hear the deep sense of disappointment in their voices as they spoke of the loss of confidence in their own abilities.

### 3.2 Pain

Like reduced comprehension, memory and concentration, headaches continued well past ‘recovery’. All participants, irrespective of age, gender or number of concussions, spoke of headaches that ‘never really went away’. Ray noted having headaches ‘24/7 in the 6‐7 out of 10 range for 2 years straight’. Ron, a 13‐year‐old boy, who no longer plays hockey, still experienced such pain in school that he sometimes had to call his parents to take him home to rest. For most, headache had become a part of everyday life:

I can just be doing simple things or hit my head on something, and I will have a headache longer then most people for the rest of the day. For most people it would wear off in an hour or so. My head never used to be sensitive to chinooks but I had migraines all this week. So, it was a super weird pain. (Marge)

The explanations received did not appear to be satisfactory to the patients:

Doctors told me that my anxiety causes my headaches, which I think they just told me cause they didn't know what was wrong with me. (Hank)

However, the adolescents themselves sometimes attributed their depression and anxiety to constant headaches.

### 3.3 Invisibility of the injury

Coping with relentless headache became an even greater burden because of the social perception of concussion: if there is no visible injury, it cannot be serious or warrant any consideration.

There's no broken bone, no cast, no scar to show what I've gone through so I hate to feel like people are sort of judging me. I have a really hard time telling people how serious it is. (Ray)

People just looked at me like you're just milking it and I'm not. (Sarah)

Interestingly, concussion is recognized only when it is manifested in obvious physical markers:

I think my last [concussion] was the only one that kinda got everyone to notice. I got physical damage because I have torn ligaments in my back, dislocated shoulder. So I had other things that people would consider ‘real injuries’. (Hank)

The appearance of normality also shows the reticence among the adolescents in expressing any pain or discomfort as a way of shielding themselves from the discovery of their condition and inviting further judgement.

### 3.4 Uncertain accommodations in school

Each participant spoke of the doubts and misconceptions in the academic environment about the concussion recovery process. While some teachers were willing to extend some assistance, many did not ‘understand’ why a student was taking so long to catch up or not feeling strong enough to do their required schoolwork.

I had a doctor's note, and the math teacher said, ‘She is fine she can write it’. I am like, I can't write it. Just three questions in and I am needing to throw up. (Marge)

My one teacher gave me extra time, told me I could do my test later. Others said I had enough information from before. (Alice)

I did try to go to a student counselor, and she tried her best, but I feel like they could have been a little more understanding of how long it takes to recover. (Sarah)

Experiences such as these concerned the older adolescents even more, as they continued to negotiate their need for accommodations while having to prove to their school administration that they were not taking undue advantage. This, for a person who is already chastising oneself for not being able to achieve standards previously met, can have significantly adverse emotional and psychological effects.

### 3.5 Mental health

‘Depression’, ‘anxiety’ and ‘fear’ are the words that appear again and again as participants speak of their continuing sense of vulnerability. These mental states are associated with the lack of control over their body and their mind. None felt prepared either by their health‐care team or coaches for these long‐term effects on their mental health following a concussion.

### 3.6 Depression

One situation that most participants cited as a cause for depression is the failure to meet their own expectations of themselves and having to accept new limitations which seem unchangeable.

If I wanted to do something or achieve something, I have always been able to do it if I try really hard. But I couldn't so that was a step back. I was pretty depressed. (Sarah)

I had a chance to go play in the WHL (Western Hockey League), I had a chance to play high level hockey, and that was my career‐ending concussion so that was a hard one to take in. I could keep up but at that level, what's keeping up and trying to drag yourself along? It was like a euphoric feeling that I was scouted. That was probably the hardest thing. (Ray)

Ray had pushed himself thinking he would be able to overcome his limitations with hard work. Eventually, he had to accept that his ‘mind and body were no longer in sync’. His voice broke and his grief was palpable as he recalled the loss of what might have been his dream profession.

Repeated injury was another reason for being depressed. Through their many injuries and concussions, participants had learned to recognize the signs. Headaches, sensitivity to light and sound, ‘personality changes’ and anxiety were indicators of yet another setback. One spoke of becoming ‘incredibly irritable’ and ‘a lot jumpy and emotional’. These changes took a toll on the parents as well, who watched fearfully as their child got hit again and again.

### 3.7 Anxiety

Anxiety is not unusual for high‐achieving youth in competitive sports or academics. When they get concussed, however, anxiety becomes both a physical and a psychological experience. With recurrent headaches and repeated injury, in addition to comprehension, memory and focus issues, anxiety seeps into every aspect of the concussed youth's life, leading to social alienation and uncertainty about what used to be routine activities.

Not being able to comprehend as well as I was before and then trying to make friends and socialize, it all goes downhill like snowplows. (Sarah)

I kind of made it through about the first half of the semester till midterms hit hard, and the stress from that really brought out the symptoms that I hadn't had in ages and it kind of put me back to that week right after I had been concussed. (Hank)

Losing financial independence was an additional source of stress for the older adolescents who had to fall back on parental support. While everyone appreciated the involvement of parents during the treatment phase, having to ask for continued help was distressing.

I never have wanted to rely on somebody else for financial help, so it hurt to have to say to my parents, Hey I can't afford gas for my car right now. (Hank)

With academic programme completion and tuition payment looming, not having a job compounded feelings of anxiety and frustration. Bartending, after isolation for 4 weeks, felt ‘massively social’ to one participant. Conversely, those who could support themselves displayed a certain degree of pride and comfort in their self‐reliance and ability to manage.

### 3.8 Fear

Another experience that was new to the youth is fear. What made this fear even more anxiety‐inducing is its inexplicability.

every time I go down the stairs I still hold on to the rail as I am walking down. I imagine myself slipping and hitting the back of my head on the stairs. (Sarah)

My greatest fear is falling. My head was like an egg. (Hank)

Apprehensions of further injury and of becoming the eponymous ‘concussion guy’ triggered fears of irreparable damage, escalating further their sense of vulnerability.

I was usually a gritty kind of guy who would go into corners with no fear but now I was scared, and every single hit I would feel dizzy and I think I had a concussion pretty much any time that I got laid out. (Ray)

Even if I am lying in bed and thinking about how many concussions I've had,… I do freak out a bit. (Marge)

These descriptions of fear were shot through with some embarrassment and a desire to laugh off what the participants seemed to see as a behavioural problem or their inability to muster the strength and courage they should have.

### 3.9 Coping

The adolescents reported coping in a number of different ways with their conditions after completing treatment and being clinically cleared to resume normal activities. The younger participants, aged between 13 and 16 years, were less forthcoming about their coping methods. They also appeared less worried about future concussions and the impact on their physical and mental health.

### 3.10 Practising alternative sports

Two out of the four participants in the age group of 17‐19 years have taken up other sports that are not entirely risk‐free but which, as they say, keep challenging their bodies and minds. These sports are ‘less aggressive’ than competitive hockey but still afford them the opportunity to ‘excel’ in some kind of performance‐based activity.

you don't wanna stop testing yourself because then you feel like there's nothing. Why am I not pushing my body to where I can actually go? (Hank)

### 3.11 Family support

Parents of younger adolescents were more directly engaged in the recovery phase of rest and isolation, making sure their children were following guidelines. Parents of older adolescents provided financial support, creating a sense of security and the opportunity for the youth to try things out. However, parents did not have much knowledge of the youths' lived fears and anxieties or how they were coping with memory loss and feelings of vulnerability.

### 3.12 Mentoring younger players

Teaching hockey to children kept the male athletes connected to the sport even though they had to recognize the loss in their own capacity. They expressed a sense of joy and satisfaction in being able to look past their present condition and fill the gap that withdrawal from competitive sport had left in their lives.

There's gotta be something better for kids, especially kids. As an adult I can think my way through, but as a kid it's confusing when your brain doesn't work and its scary. (Ray)

Knowing that concussion knowledge among athletes is often anecdotal, informal and obtained from non‐specialized sources, the adolescents use their research to inform younger players of the risks of injury and ways of avoiding and responding to concussion.

### 3.13 Developing self‐awareness

In the years of coping with the impact of concussion, the athletes experienced a dearth of clear, consistent and knowledgeable messages from their coachers and teachers. Information from health‐care providers has also not been as comprehensive in explaining the post‐concussion condition. The assessment tests they completed did not capture the losses they were struggling with. In fact, some noted that they ‘got good at the tests’. They managed to pass these tests, but ‘there are lots of things that are going on still’.

The older adolescents had a greater experiential sensitivity to the risk of repeated concussions. Although part of this sensitivity stems from a fear of narrowing options and a pessimistic sense of one's susceptibility to more injury, it makes them averse to jeopardizing their physical and mental health.

More than anything, my grades took a hit. I am scared that the next one might make it worse or do some serious damage to my brain. I know it's a matter to time if I keep going down this road, it's gonna happen eventually. I can't keep escaping it every time. (Marge)

The impact range of concussion has also brought about the realization that suffering in silence is neither prudent nor possible.

I was trying coping mechanisms and it wasn't working. I was at the gym and feeling so bad, and I was kind of at the lowest point, so I had a conversation with Ray and my mum and was kind of hey, you know, something like really might be wrong, and I kind of always denied to myself I was feeling bad… it was finally a sort of breakthrough, something really bad is going to happen if I don't get help here. (Hank)

In addition to opening up to peers and family, the older athletes took the initiative to teach themselves more about concussion. The information they gathered obviously did not answer all their questions, but it made them aware that their journey may be a long one and they would have to come to terms with it in their own way.

I've learned most of my coping mechanisms in the last year which is pretty sad because I've had concussions for the last 7 years. (Hank)

Others avoided research for some of the same reasons. Concussion stories report dire scenarios. ‘I try not to freak myself out’, said one participant. Instead, she focused on extensive pain relief measures, such as chiropractic, physiotherapy and massages.

### 3.14 Mental toughness

The youth relied to a great extent on their mental strength to get them through. Among the younger group, the main concern was the loss of opportunity for a higher level of performance in sports. One 13‐year‐old boy said that although he had had three concussions, he was not afraid of getting hurt again. He reluctantly wears a ‘concussion padding’ at the insistence of his parents:

Hockey is the sport, supposed to be contact, risks are known ahead of time. (Devon)

Among the older group, awareness of coping challenges is more acute. One participant explained his coping strategy in a striking way by invoking his experience with severe bullying and abuse in school:

I had a teacher that ended up getting fired for abuse, you got gangs and all that stuff. And if you're vulnerable they'd go for you. You always have to appear tough. I built the most mental toughness out of that. That's when I had some pretty major depression. Getting over that has helped me get over anything involved with this. (Ray)

By ‘this’, Ray did not mean only the pain or injury of concussion. Rather, by comparing the trauma of being bullied with the impact of concussion, he expressed his determination in not allowing the sense of alienation and distress associated with feeling impaired to overcome him.

Basically, being put down no matter what. Being scared to openly ask or getting shut down. You've been shut down so much. I think the concussion has definitely contributed to that. Not fear of rejection but fear of not being seen as equal.

For Ray, mustering the courage to look beyond the concussion, as he did with the bullying, has kept him going. He needs to continually look at what he describes as ‘the big picture – this is what I have, this is what I've got, let's see what I can make of it now’.

### 3.15 Friendship and understanding

All participants clearly expressed the positive impact of meaningful and caring communication with peers as surpassing the role of family in the recovery process. The understanding of peers made them ‘feel less stressed’, and was even empowering when they were able to share their experience:

I feel like Ray has gone through a lot of similar stuff though not the same. He feels differently than I do but he's somebody I can talk to and he'll have answers a lot of the time. (Hank)

Sarah explained the critical role of friendship in the recovery process and the ability to regain normalcy. Relating both her agonizing and positive experiences with acceptance, she captured the powerful impact of peer relationships on a concussed individual's sense of self:

It's hard to find friends when you have that issue in your head, when you're trying to listen to people and trying to comprehend it as fast as you can. I used to make like good comebacks and jokes and stuff. Good conversations but they take a moment to come out like minutes too late. Or just say things like ‘ok, wait what? That doesn't make sense?’ That was hard. Still hard today… It's like do you focus on yourself or do you try to focus on others? But then it's hard to focus on others when you can't really think

With the only close friend she had left, Sarah was able to connect and share.

parents are nice to talk to, you enjoy that, but having a friend that's there to talk to and to support you all the time, it's nice. And it makes you feel a lot better… With my social anxiety, it makes me feel better.

## 4 DISCUSSION AND CONCLUSION

The purpose of this study was to explore the experience of adolescents in their recovery from concussion. Although limited in scope by programme timeline and available resources, the study provided valuable data on what recovery meant to the adolescents and how they managed to move through their so‐called recovered state with pain, isolation, anxiety and depression to make sense of their ‘new normal’. Some differences were noted between the younger and older adolescents (Table 2). However, more targeted research is needed to understand the emotional and psychological impact of concussion among pre–high school athletes.

**TABLE 2:** Age‐related differences

	Age‐related differences	
	13‐16	17‐19
Isolation during treatment	Boredom; resentment at parental oversight; fear of being dropped from team	Appreciation of family support; fearful of mental health; aloneness
Social acceptance	Easy acceptance; help from peers in catching up with sports and studies	Difficulty with communication; alienation; incredulity of peers
Memory loss	Loss in the immediate aftermath of injury	Prolonged loss and sense of permanent damage
Knowledge of concussion	Dependence on parents and physicians; not clear on why or how protocol works	Involvement in decisions through consultation with family and physicians

Several factors emerged as critical in the adolescent experience of concussion, most important among them is the limited concussion knowledge and training available to athletes, coaches and teachers. The concussion knowledge our participants appeared to have was minimal even though they were playing competitive sports, some at a fairly high level. Although not everyone went back with severe symptoms, all returned to school and play in uncertain conditions, assuming they would be ‘safe’. Despite having had a previous concussion, players often continued playing not knowing that they had suffered a second or third concussion.

A similar lack of awareness and training was evident in the athletes' experience in school, where some teachers were ‘generous’ and provided accommodations while others were non‐supportive. Understanding the range of problems concussed young athletes may experience in return to learning is still inadequate and protocols are applied inconsistently.^22^, ^23^ ‘Limited research’ on the effects of concussion on young learners and ‘inadequate training on concussion management’ in the school setting contribute to the difficulties faced by youth on returning to school after a concussion.^22^ Students had to figure out how to gain credibility, make up missed work and at the same time manage their continuing symptoms. Even in places where legislation and policies exist to address concussion safety and ease of return to school after a concussion, implementation remains problematic. The protocols are applied only to physical education and responsibility is placed only on ‘relevant’ school staff.^1^ Even in its revised and updated version, the Education Act of Ontario, for example, directs concussion training and awareness to sports‐related activities conducted by school boards in the province^24^ rather than the full range of a concussed student's life in school. A team‐based approach, although difficult to organize consistently with variable resources,^22^ may be best equipped to assist a student with prolonged symptoms. Such a team would be able to provide the flexible and individualized accommodations that such students need.^25^

Another area of concern that remains relatively under‐researched is the mental health implication of concussion.^26^, ^27^ The older youth in our study were candid in their expressions of the mental health challenges they faced on a daily basis. The inability to comprehend a conversation, in addition to the incredulity of peers, can create immeasurable uneasiness and apprehension among youth, more so when they cannot explain the nature of their problem. Various levels of psychological and emotional pressures result from the disruption of identity, the stress of trying to maintain normalcy, the lack of understanding from peers in school and sport, and giving up the sport the players cherish and excel at.^3^, ^4^, ^28^ In addition, difficulties with remembering and retaining information, the need to rehearse a conversation before engaging in it and the feeling of being trapped within a label of injury persist. The invisibility of the injury and the sense being less than their pre‐concussion selves lead to an exacerbation of emotional loneliness and, consequently, depression. While the younger children were anxious and ready to get going, the four young adults felt that they might have been able to cope better with more timely and targeted information about the cognitive and emotional impact of concussion. Thus, patient education about mental health stresses following concussion was emphasized as a required part of the treatment and follow‐up processes.

In the face of these challenges, the adolescents have adapted, coped and continue to move forward on the path of ‘resilience’. Leipold and Greve^29^ make a distinction between coping and resilience. Coping is seen as short‐term ways of managing emotional and situational pressures. Resilience is an interactive process that grows out of several types of coping strategies, leading to a place where long‐term positive change can occur. As Mainwaring's research showed,^30^ the desired outcome that drives and motivates players through the rehabilitation process is the ‘Restoration of Self’, which means ‘overcoming the obstacles, challenges, and consequences of physical disability and restoring one's lifestyle in all domains of functioning’ (145). Similarly, among our participants, the older youth have not only used their available resources—such as family, work and school—to be able to function within their normal spheres of activity, but they have also made changes to their life that may enable them to reconstruct their identities. By withdrawing from the sport that is the cause of their multiple injuries and accepting their reduced mobility, the athletes have shown their ability to adapt. The narrative of one person who used his experience of coping with abuse as a way to reflect upon and develop strategies to fight his feelings of alienation and depression is a striking example of resilience. Becoming more alert and cautious about their own safety and that of their teammates, finding ways of incorporating their knowledge and awareness into educating others, and trying to work on their self‐reliance are all important signs of the ability of these young people to look forward to stability and improvement in physical and mental health. This effort needs to be recognized, validated and supported actively.

## 5 LIMITATIONS

Our study has some limitations. First, this being an internship research project, we were limited both by the time available to complete the study and by the resources to expand our recruitment. As mentioned earlier, the number of our participants was small and the demographic was fairly diverse in age, severity of injury, gender and sport of injury. Second, the small number of our participants may not have allowed us to fully develop a theory of adolescent patient experience of recovery from concussion; however, the narratives, in detail and directness, have revealed a clear gap between the medical standard of recovery and the adolescents' goals and expectations of recovery. Third, these findings may not represent the experiences of those patients who have recovered completely, have received targeted accommodations in school or have been able to return to play without any symptoms.

## AUTHOR CONTRIBUTIONS

Ash Kolstad: Co‐designed the study and collected and analysed the data. Vishvesh Prajapati: Co‐designed the study and collected and analysed the data. Gina Samuel: Co‐designed the study and collected and analysed the data. Keith Owen Yeates: Sponsored internship through the Integrated Concussion Research Program, University of Calgary; collaborated in study design and data analysis.

## Data Availability

The data that support this study are not openly available due to consideration of patient confidentiality. However, findings of this study may be made available without identifying information on request from the corresponding author.
